# Diet Switching by Mammalian Herbivores in Response to Exotic Grass Invasion

**DOI:** 10.1371/journal.pone.0150167

**Published:** 2016-02-26

**Authors:** Carolina Bremm, Paulo C. F. Carvalho, Lidiane Fonseca, Glaucia A. Amaral, Jean C. Mezzalira, Naylor B. Perez, Carlos Nabinger, Emilio A. Laca

**Affiliations:** 1 Grazing Ecology Research Group, Federal University of Rio Grande do Sul, Porto Alegre, Brazil; 2 Embrapa Southern Region Animal Husbandry, Bagé, RS, Brazil; 3 Department of Plant Sciences, University of California Davis, Davis, California, United States of America; College of Charleston, UNITED STATES

## Abstract

Invasion by exotic grasses is a severe threat to the integrity of grassland ecosystems all over the world. Because grasslands are typically grazed by livestock and wildlife, the invasion is a community process modulated by herbivory. We hypothesized that the invasion of native South American grasslands by *Eragrostis plana* Nees, an exotic tussock-forming grass from Africa, could be deterred by grazing if grazers switched dietary preferences and included the invasive grass as a large proportion of their diets. *Bos taurus* (heifers) and *Ovis aries* (ewes) grazed plots with varying degrees of invasion by *E*. *plana* in a replicated manipulative experiment. Animal positions and species grazed were observed every minute in 45-min grazing session. Proportion of bites and steps in and out of *E*. *plana* tussocks were measured and used to calculate several indices of selectivity. Both heifers and ewes exhibited increasing probability of grazing *E*. *plana* as the proportion of area covered by tussocks increased, but they behaved differently. In agreement with expectations based on the allometry of dietary preferences and morphology, ewes consumed a low proportion of *E*. *plana*, except in areas that had more than 90% *E*. *plana* cover. Heifers consumed proportionally more *E*. *plana* than ewes. Contrary to our hypothesis, herbivores did not exhibit dietary switching towards the invasive grass. Moreover, they exhibited avoidance of the invasive grass and preference for short-statured native species, both of which should tend to enhance invasion. Unless invasive plants are highly palatable to livestock, the effect of grazing to deter the invasion is limited, due to the inherent avoidance of the invasive grass by the main grazers in the ecosystem, particularly sheep.

## Introduction

Threats to the sustainability of grassland production of multiple ecosystem services require that we investigate the factors that drive community dynamics beyond short-term productivity. It is necessary to design management strategies with productivity sustained by the control or reversal of invasion by undesirable exotic plants. This requires basic knowledge of factors that determine the ability of invasive species to displace other species and species-specific herbivore pressure in plant communities that contain species with contrasting density, nutritional quality and palatability [1]. For example, in northern Europe, the positive effect of moderate grazing by cattle on plant community has been used to help restore species-rich grasslands [2].

The invasion and dominance of exotic plant species has reduced native species diversity of plant communities in many ecosystems [3, 4, 5, 6]. The ability of invasive plants to become dominant depends on their interactions with native vegetation composition, grazing intensity, herbivore body size, plant productivity and climatic variability [7, 8, 9]. Herbivore selectivity of grazing patches, associated with other disturbances (e.g. resource availability, environmental factors), have resulted in dominance of more tolerant plant species in grassland ecosystems [10, 11]. [12] found predominance of different functional groups depending on grazing intensity, with higher contribution of “conservative” (tall grass) and “capture” (short grass) strategies in low and high grazing intensities, respectively.

Diet switching, the process by which animals consume abundant prey species disproportionately more than less abundant ones, can stabilize community composition [13]. Community composition is stabilized by this process because species present in low relative abundance are avoided, and species present in high proportion are selected by the consumer. When relative abundance of invasive plants increases grazing might impede proliferation of the invader if herbivores switch from avoiding to selecting the invasive plant in a “diet switching” pattern associated with maximizing intake [14].

*Eragrostis plana* Nees, an invasive exotic grass, has invaded the native grasslands of Campos Biome in South America. This exotic plant can persist for many years and it is listed among the worst of threats currently facing these grazing lands [15]. *E*. *plana* is a tufted warm season (C4) perennial grass originating from South Africa with low palatability and is generally avoided by grazing animals. Thus, degradation by high grazing intensities [15] and animal type used for grazing (mainly cattle [16]), are the major management factors favoring *E*. *plana* dominance in native grasslands.

Herbivore body size can be important to explain the magnitude and scale of grazing effects on the abundance of dominant plants [10, 17]. Different mammalian herbivores have distinct behavioral strategies and ability to overcome constraints on preferences imposed by forage abundance and spatial heterogeneity [18, 19]. Cattle and sheep perceive and respond to heterogeneity in their environments in different scales of perception [20]. Sheep are better able to buffer changes in forages available and to maintain their diets closer to the preferred proportions than cattle as available proportions of different forages deviate from preferred ones. Cattle have potential to reduce the abundance of dominant plants [21, 22], because they can utilize more fibrous foods, while sheep are more capable of selecting less abundant food items of higher quality [23]. Differences in scales of perception between different mammalian herbivores that might affect selectivity of one food option over another could be used to design site-specific grazing strategies aiming to deter exotic grass invasion, but the mechanistic bases for such management remain poorly understood.

Our study focuses on the relationship between the dietary preferences by two mammalian herbivores that are common—cattle and sheep—and the degree of invasion by an exotic grass. We hypothesize that the invasion of native South American grasslands by *E*. *plana*, an exotic tussock-forming grass, could be deterred by grazing if grazers switched dietary preferences and included the invasive grass as a large proportion of their diets. We predict that both herbivores will increase proportion of *E*. *plana* in the diet as it becomes more dominant, but sheep will be able to maintain greater selectivity for short grasses than cattle as the proportion of *E*. *plana* increases.

## Materials and Methods

### Ethics statement

The study was approved and carried out in strict accordance with the recommendations of the Ethical Review Committee on the Use of Animals of the Federal University of Rio Grande do Sul, Brazil (project n° 20521). The experimental animals were conducted in conformity with the national and international guidelines and standards, especially the Law 11.794 of November 8, 2008, which governs the creation and utilization of animals in teaching and research activities.

### Experimental design and treatments

We studied diet selection by sheep and cattle grazing plots with various proportions of the exotic invasive grass *Eragrostis plana* Nees, which is a tussock-forming warm-season (C_4_) perennial grass from South Africa. The experiment was carried out at the Research Station of the Federal University of Rio Grande do Sul, Brazil (30°05’51”S, 51°40’42”W) in November and December 2009. The climate is subtropical humid (Cfa classification, Köppen), with annual precipitation of 1440 mm, well distributed throughout the year; June is the wettest month (168.2 mm), and December is the driest (97.7 mm).

Eight plots were selected from a large native grassland area with spatially variable density of *E*. *plana*, such that two plots had each of four nominal levels (treatments) of *E*. *plana*: 0, 25, 50 and 75% cover. Plots were selected by mapping the cover of *E*. *plana* in each square meter of two areas totaling 2,000 m^2^ and then positioning the plots on the map to obtain the desired treatment. *E*. *plana* tussock cover was visually estimated following methodology of [24]. Area not covered by tussocks was inter-tussock. In order to obtain proportions of tussocks as similar as possible to the nominal treatment values, excess of tussocks was removed with a hoe and the affected areas were replaced with inter-tussock sod (short native grasses) obtained from neighboring areas one year prior to the grazing tests.

Plot sizes were chosen to include the same absolute area of inter-tussock vegetation (100 m^2^) in all treatments. Thus, plot areas were 100, 133, 200 and 400 m^2^ for treatments with 0, 25, 50 and 75% of average tussock cover at the whole-plot or paddock level. We maintained a constant area of the inter-tussock to avoid confounding the effect of proportion of tussock cover with the effect of decreasing absolute availability of inter-tussock. This emphasizes the effect of tussocks as potential deterrents of selection for the preferred inter-tussock, instead of the effect that an increasing proportion of tussock cover has through the concomitant reduction of inter-tussock. The effects of tussock cover in this experiment are not due to reductions in the absolute availability of the alternative inter-tussock.

**Fig 1:**
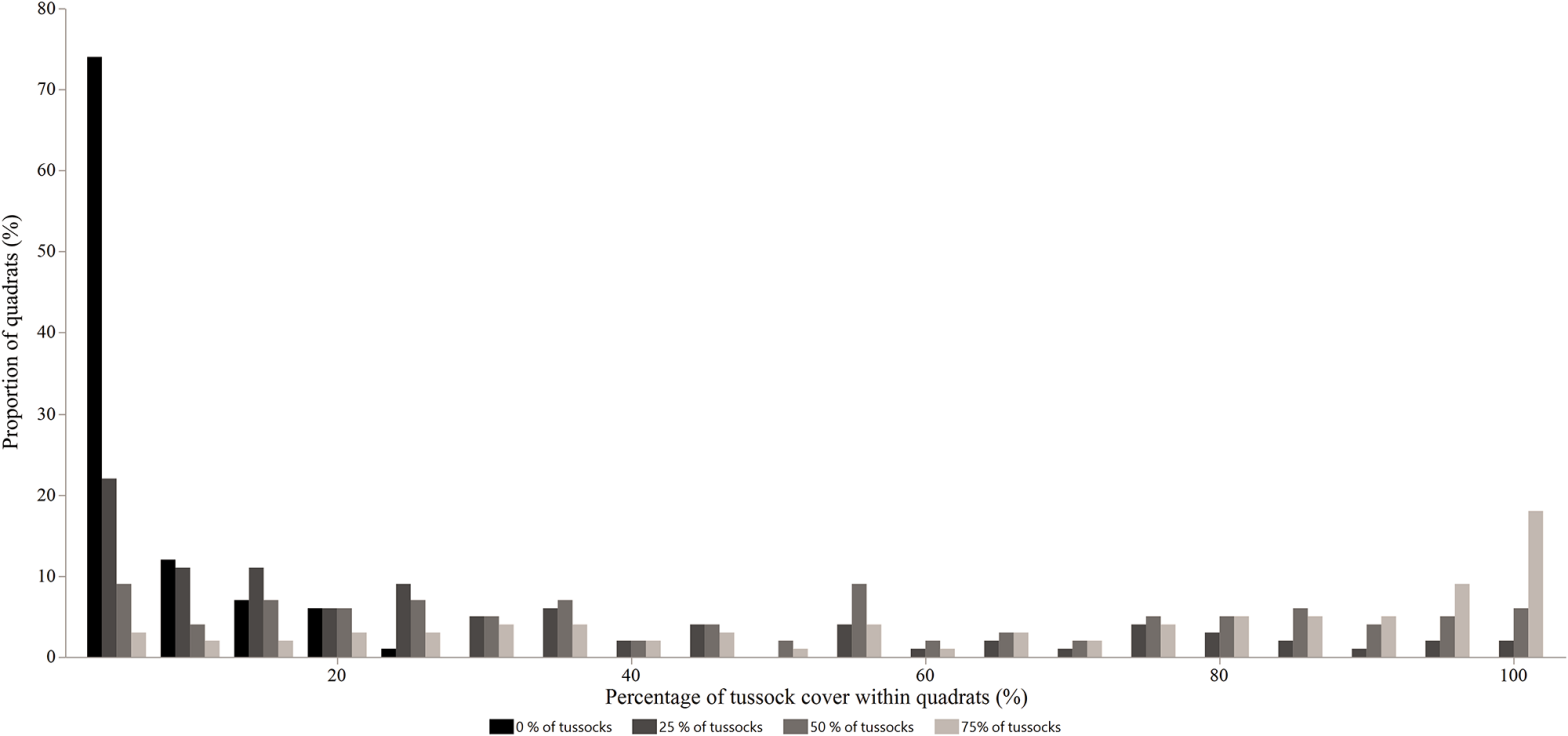
Histograms of tussock cover measured in a 1-m^2^ grid in each of the treatments applied at the whole plot or paddock level (0, 25, 50 and 75% of *Eragrostis plana* Nees.) Spatial variability in tussock cover resulted in a variety of values observed within each treatment.

Due to natural spatial variability, tussock cover in each 1 m x 1 m square varied widely within each plot (Fig 1). As described in detail below, diet selection of animals was measured at each 1 m x 1 m square and then related to *E*. *plana* cover both at plot-level (treatment, categorical explanatory variable) and at feeding site level (% *E*. *plana* cover, continuous explanatory variable).

Each plot was grazed once by a group of four heifers for a total of 8 grazing tests but due to a mistake, animal positions were not recorded in two of the tests. For ewes’ evaluation, each plot was grazed twice by a group of six ewes for a total of 16 grazing tests. Due to time constraints, only two plots were grazed and observed per day, one in the morning (AM block) and one in the afternoon (PM block) in one period for heifers and in two periods for ewes (different days). The order of plot grazing within time of day and day of evaluation was randomized for each animal species. The grazing tests with heifers were conducted before the tests with ewes. Herbivore species were tested in separate experiments because it was not logistically possible to have both species available for experimentation at the same time.

Duration of each grazing test was approximately 45 min, because preliminary measurements in adjacent areas similar to the experimental plots indicated that inter-tussock height would not be reduced more than 10% in 45 min. Inter-tussock height was maintained at approximately 11.4 cm for cattle and 9.5 cm for sheep throughout grazing tests, which according to [25] should allow non-limiting intake rate for both animal species. Therefore, plots were grazed with a low stocking rate (low total demand of forage) but with a high stocking density (high rate of forage demand) relative to typical management.

### Sward characteristics

Botanical composition was determined prior to grazing tests using the inclined point quadrat method [26] with 150 observations in each plot. Inter-tussock areas were composed by grass (average ± standard deviation)—*Andropogon lateralis* (30.7 ± 2.6%), *Paspalum notatum* (21.3 ± 5.3%), *Axonopus afinnis* (18.5 ± 12.0%), *Paspalum umbrosum* (4.7 ± 3.9%), *Cynodon dactylon* (3.5 ± 2.2%), *Coelorachis selloana* (3.5 ± 2.2%), *Setaria parviflora* (2.9 ± 4.1%), *Paspalum nicorae* (2.5 ± 3.5%), *Kyllinga odorata* (2.5 ± 3.5%) and *Dichanthelium sabulorum* (1.0 ± 1.4%)—and the legume *Desmodium incanum* (9.1 ± 4.8%). Tussock vegetation consisted of *E*. *plana* in vegetative phenological stage. The vegetation found inside the tussocks (intra-tussock, 8.7 ± 2.9%) was predominantly *Desmodium incanum*. The inter-tussock and tussock vegetations were separated on a horizontal plane, while the intra-tussock areas existed in the same space as the tussocks, separated on a vertical plane.

Inter-tussock height was measured at 100 points and tussock height was measured at 50 points before and after grazing using a sward stick [27]. Aboveground biomass was determined by clipping six quadrats (0.5 m x 0.5 m; three in tussock and three in inter-tussock areas) per plot at ground level using a hedge-trimmer before grazing tests. Herbage samples were weighed fresh and then oven dried at 60°C for 72 h to determine dry matter (DM) content.

### Animals

Four crossbred heifers (Angus x Brahman) weighing 286.7 ± 1.2 kg and 12 adult Suffolk ewes weighing 51.0 ± 0.72 kg were used. Ewes were separated into two groups of six animals, each consisting of four “tester” animals and two “companion” animals to reduce behavioral artifacts due to small group size [28]. Each group of six ewes grazed each plot once in the morning and in the afternoon depending on its block. This resulted in a total of eight grazing tests for each ewe’s group. [29] provided details about the experimental procedure.

Both ewes and heifers grazed an adjacent area with similar botanical composition and sward structure for approximately 30 days immediately before the experiment to become familiarized with observers, recording equipment and the experimental procedure.

### Diet measurements

Animals were observed during periods of peak grazing activity (after 8:00 and 18:00 h, [30]). Animal position and stratum grazed (tussock, inter-tussock or intra-tussock) were recorded for each animal every minute. Location was recorded on a scaled map of each plot with a 1-m^2^ grid that matched the grid used for vegetation measurements. Animals were not fasted prior to the grazing tests in order to avoid impacts on diet selection [31].

We analyzed (i.e., statistically modeled) four response variables for animals: (1) probability of biting tussock at the 1-m^2^ level, (2) probability of grazing intra-tussock at the 1-m^2^ level and selectivity index at the (3) path and (4) feeding station to bite levels. Probability of grazing tussock represents the probability that an animal grazes tussock when observed in any given minute. We used a selectivity index defined as the proportion of a stratum of forage in the diet divided by the proportion of that stratum in the herbage available [32]. We calculated partial selectivity [20] at each of two spatial scales (patch and integrated from feeding station to bite). Partial selectivity at the plot level was the proportion of inter-tussock stratum in the animal’s path (squares visited and their 8 neighbors) divided by the proportion of inter-tussock stratum in the plot. Partial plot-level selectivity reflects the selection at the foraging path level. Partial selectivity from feeding station to bite level was the proportion of observed bites in inter-tussock and intra-tussock strata divided by the proportion of inter-tussock stratum in each square visited. Selectivity values > 1.0 (< 1.0) indicated that inter-tussock was selected more (less) than expected by random encounter.

### Statistical analysis

Statistical analyses of diet variables were performed separately for heifers and ewes. For the analyses of probability of grazing tussock and intra-tussock, observations were grouped in 11 classes of % tussock cover within each 1-m^2^ (0, 1–10, 10–20, …, 90–100) and each class was characterized by its average % tussock. Residuals were visually inspected to assess the presence of trends and heteroscedasticity. Heteroscedasticity was detected in one case and corrected by using generalized least squares. Data were analyzed using a linear model with a logit link, called “logistic regression”. Modeling started with a full model expressed as a generalized linear mixed model (glmer) for binomial distribution that was later simplified according to the scheme of [33]. The full model for probability of grazing tussock or intra-tussock included fixed effects for percentage of tussock cover in the whole plot (categorical treatment), percentage of tussock cover in the 1 m^2^ grazed (continuous covariate), time of day (AM vs. PM) and their interactions. Random effects were grazing session and individual animal. Probability of grazing intra-tussock by heifers was low (0.012 ± 0.009), and it was not analyzed. Probability of grazing tussock and intra-tussock by ewes were analyzed with fixed effects for percentage of tussock cover in the plot, percentage of tussock cover in the 1 m^2^ grazed and their interaction, and with random effects for animal and for the interaction grazing test by animal.

The full model for selectivity included fixed effects for percentage of tussock cover in the plot (categorical treatment), time of day (AM vs. PM), selectivity level (path vs. smaller scales) and the interaction of selectivity level and treatment. A random effect for animal was included. Vegetation characteristics were analyzed with fixed effects for percentage of tussock cover in the plot and the time of day (AM vs. PM). We used R 3.2.2 software [34].

## Results

### Sward characteristics

There were no significant differences (*P* > 0.05) in sward height or aboveground biomass of either inter-tussock or tussock strata among treatments (Tables 1 and 2). Average sward height of inter-tussock and tussock strata were 10.3 ± 0.3 and 40.4 ± 1.1 cm for heifers and 10.5 ± 0.3 and 44.2 ± 1.3 cm for ewes. Inter-tussock sward heights were considered not to limit intake rate of either cattle or sheep. According to the equation defined by [25] the observed sward heights could provide 99.1% of the potential intake rate for sheep and 99.0% of the potential intake rate for cattle. Mean aboveground mass for inter-tussock and tussock were 2.9 ± 0.2 and 20.4 ± 1.9 ton DM⋅ha^-1^ for heifers and 2.3 ± 0.4 and 18.9 ± 1.4 ton DM⋅a^-1^ for ewes. Actual tussock cover in the four treatments were 2.5 ± 0.3, 26.6 ± 1.7, 45.8 ± 1.6 and 69.8 ± 1.2 percent.

**Table 1:** F tests for terms in the model for sward grazed by heifers.

Sward characteristic	Effect	Degrees of freedom	F	P(>F)
Inter-tussock height	% Tussock at plot-scale	3	0.854	0.4988
Tussock height	% Tussock at plot-scale	2	2.638	0.1400
Aboveground mass for inter-tussock	% Tussock at plot-scale	3	0.191	0.8997
Aboveground mass for tussock	% Tussock at plot-scale	2	1.093	0.3861

**Table 2:** F tests for terms in the model for sward grazed by ewes.

Sward characteristic	Effect	Degrees of freedom	F	P(>F)
Inter-tussock height	% Tussock at plot-scale	3	2.819	0.0882
Tussock height	% Tussock at plot-scale	2	1.649	0.2513
Aboveground mass for inter-tussock	% Tussock at plot-scale	3	0.989	0.5032
Aboveground mass for tussock	% Tussock at plot-scale	2	1.637	0.3792

### Diets

**Fig 2:**
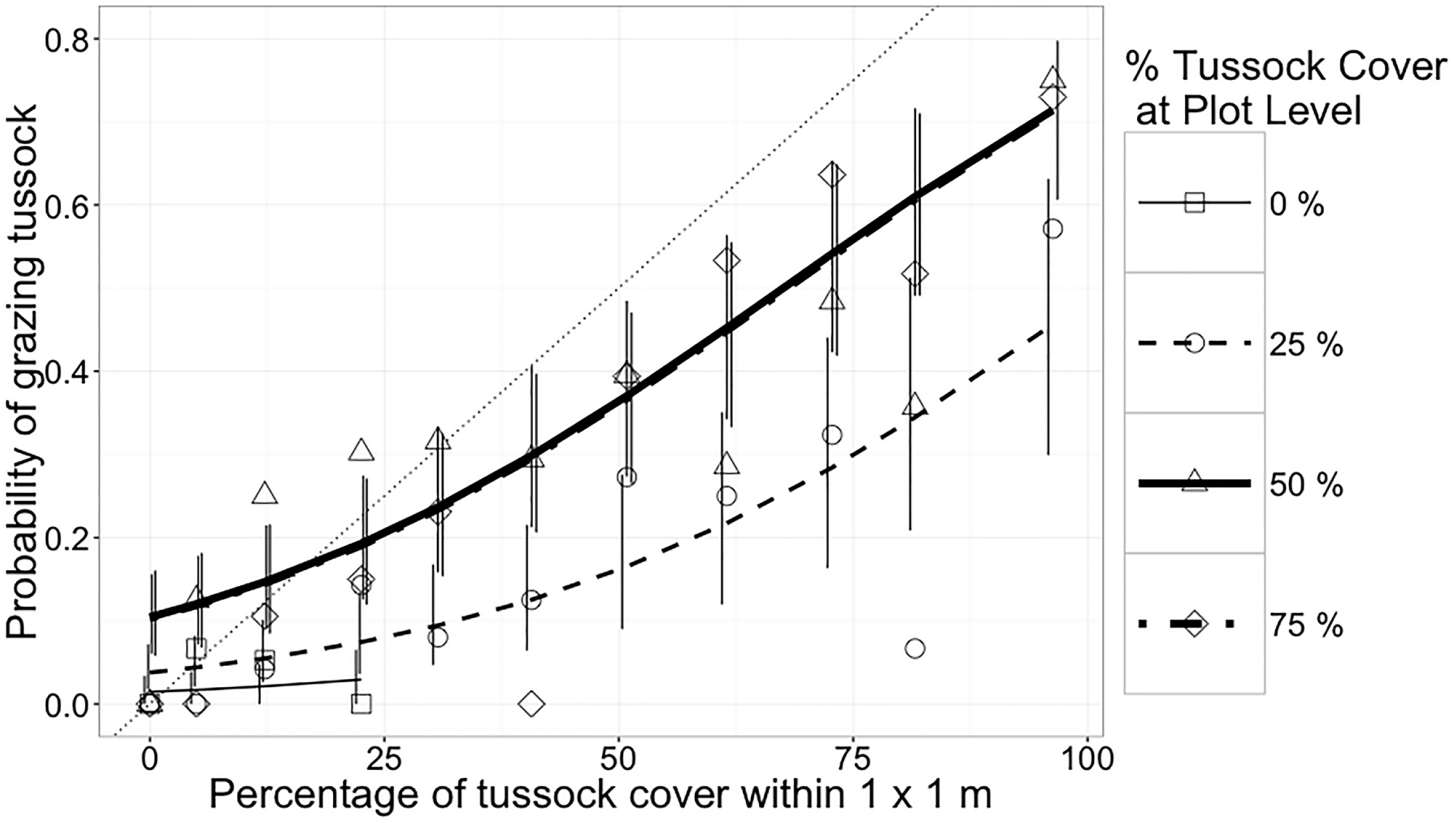
Probabilities of grazing tussocks (mean and 90% confidence interval) by heifers as a function of treatment (nominal tussock cover at the plot level) and tussock cover in each 1-m^2^ cell grazed (n = 1,132). The thin dotted line shows the no-selection expectation where probability of grazing tussock equals the proportion of area covered by tussock. Note that lines for 50 and 75% plot-level tussock cover are almost identical. Vertical lines are 90% confidence intervals. Symbols are raw proportions calculated by pooling data across animals, time of day and sessions.

For heifers, proportion of bites on tussocks increased with increasing proportion of tussocks at the feeding station in all treatments (Table 3). At any level of small-scale (1 m^2^) tussock cover, probability of grazing tussock increased with increasing large-scale (plot-level) tussock cover, except for treatments with 50 and 75% tussock cover, which exhibited almost identical responses (Fig 2).

**Table 3:** Wald’s Chi-sq tests for terms in the model for probability of biting tussock in heifers.

Effect	Chi-sq	Degrees of freedom	Pr(>Chisq)
% Tussock at plot-scale	15.52	3	0.0014
% Tussock at 1 m^2^ scale	138.48	1	<0.0001

**Fig 3:**
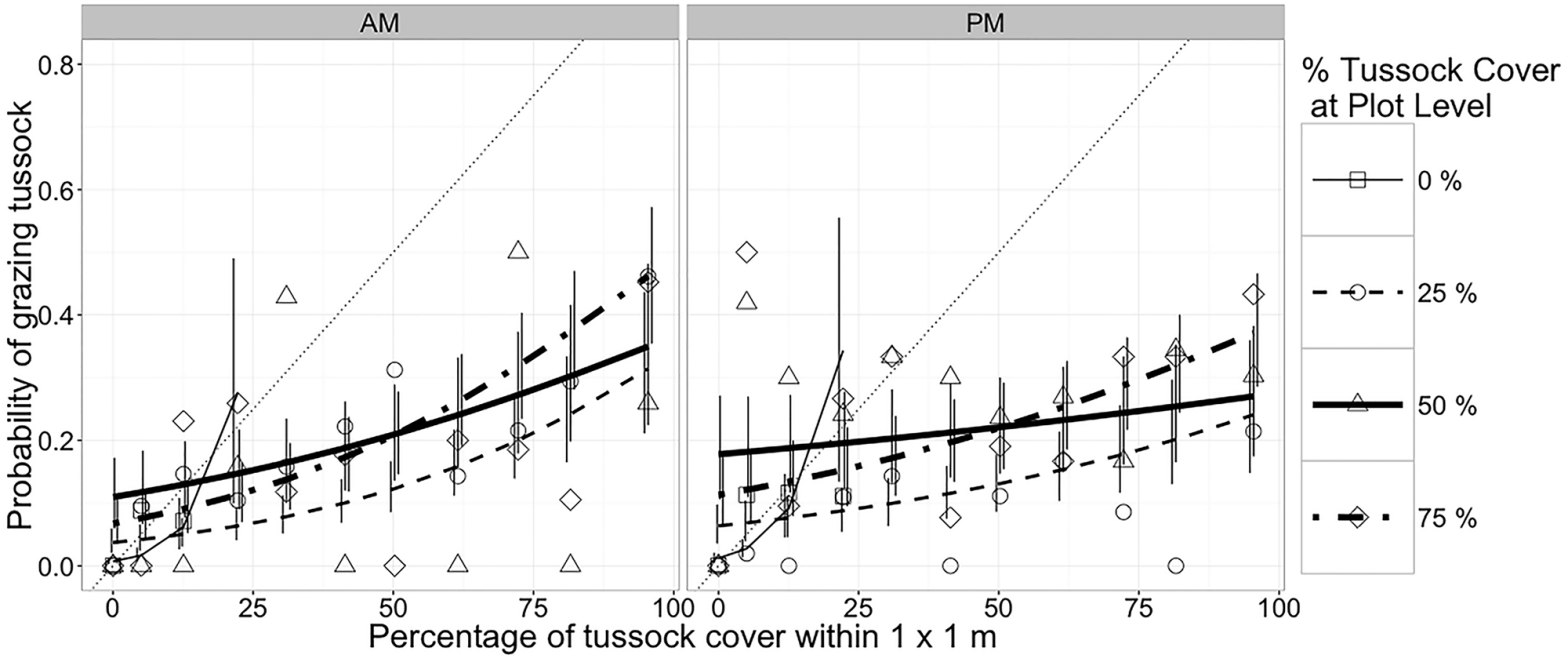
Probabilities (mean and 90% confidence interval) of grazing tussocks by ewes in morning (AM) and evening (PM) sessions as a function of treatment (nominal tussock cover at the plot level) and tussock cover in each 1-m^2^ cell grazed (n = 2,217). The thin dotted line shows the no-selection expectation where probability of grazing tussock equals the proportion of area covered by tussock. Vertical lines are 90% confidence intervals. Symbols are raw proportions calculated by pooling data across animals, time of day and sessions.

The effect of tussock cover in the 1 m^2^ grazed on the probability of tussock grazing by ewes differed between treatments (*P* < 0.001; Table 4). The probability of grazing tussock increased with increasing large-scale (plot-level) tussock cover, but with less intensity in treatment with 50% tussock cover (Fig 3). The interaction between the effect of tussock cover in the 1 m^2^ and time of day was also significant (*P* = 0.0172; Table 4). The ewes were more likely to graze on tussocks in the morning than in the afternoon (Fig 3).

**Table 4:** Wald’s Chi-sq tests for terms in the model for probability of biting tussock in ewes.

Effect	Chi-sq	Degrees of freedom	Pr(>Chisq)
% Tussock at plot-scale (T)	13.45	3	0.0038
% Tussock at 1 m^2^ scale (X)	23.55	1	<0.0001
Time of day (AM-PM)	0.28	1	0.5943
Interaction T x X	21.96	3	<0.0001
Interaction AM-PM x X	5.68	1	0.0172

**Fig 4:**
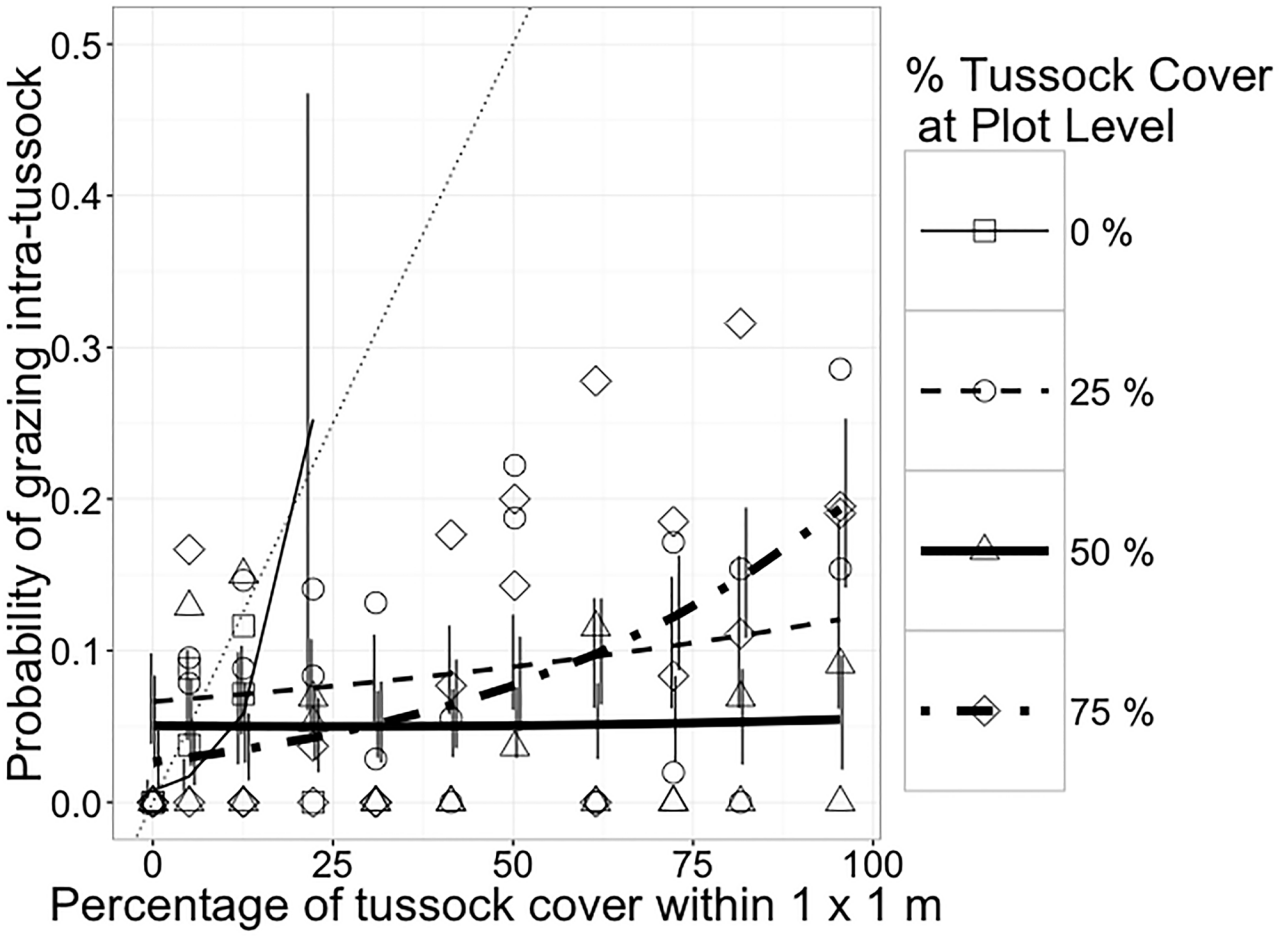
Probabilities (mean and 90% confidence interval) of grazing intra-tussocks by ewes as a function of treatment (nominal tussock cover at the plot level) and tussock cover in each 1-m^2^ cell grazed (n = 2,217). The thin dotted line shows the no-selection expectation where probability of grazing tussock equals the proportion of area covered by tussock. Vertical lines are 90% confidence intervals. Symbols are raw proportions calculated by pooling data across animals, time of day and sessions.

Ewes didn’t replace inter-tussock grazing with tussocks grazing. As percentage of tussock cover at 1 m^2^ scale increased, ewes increased the probability of grazing the intra-tussock legumes in all treatments (*P* < 0.001; Table 5), with higher increasing at 75% tussock cover (Fig 4).

**Table 5:** Wald’s Chi-sq tests for terms in the model for probability of biting intra-tussock in ewes.

Effect	Chi-sq	Degrees of freedom	Pr(>Chisq)
% Tussock at plot-scale (T)	15.09	3	0.0017
% Tussock at 1 m^2^ scale (X)	17.21	1	<0.0001
Interaction T x X	23.70	3	<0.0001

### Selectivity across scales

**Fig 5:**
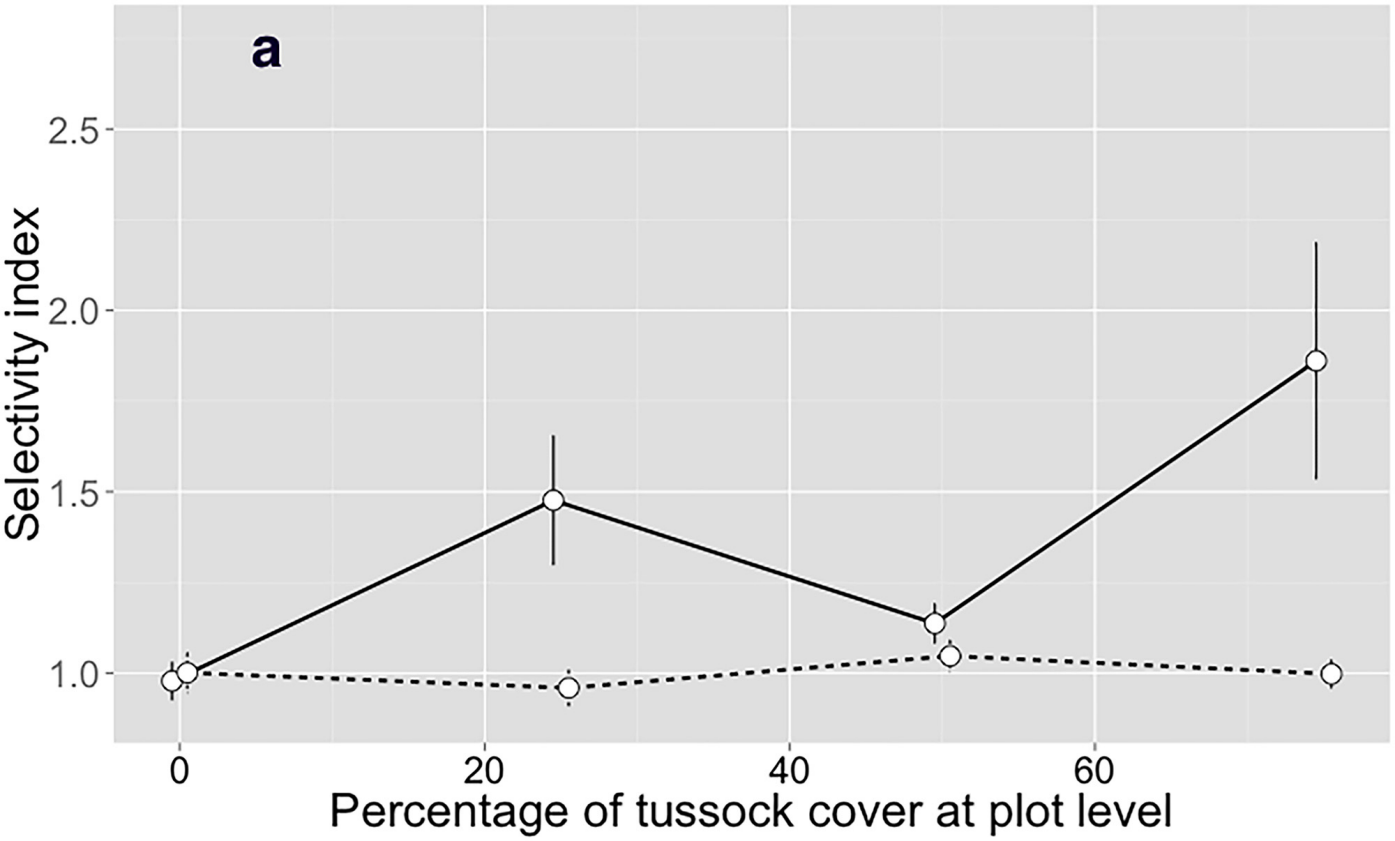
Partial selectivity levels (mean ± standard error) for inter-tussock stratum by heifers. The continuous line represents the feeding station to bite scale and the dashed line represents the search path or patch scale. Values >1.0 mean that the inter-tussock was selected more than expected by random encounter.

**Fig 6:**
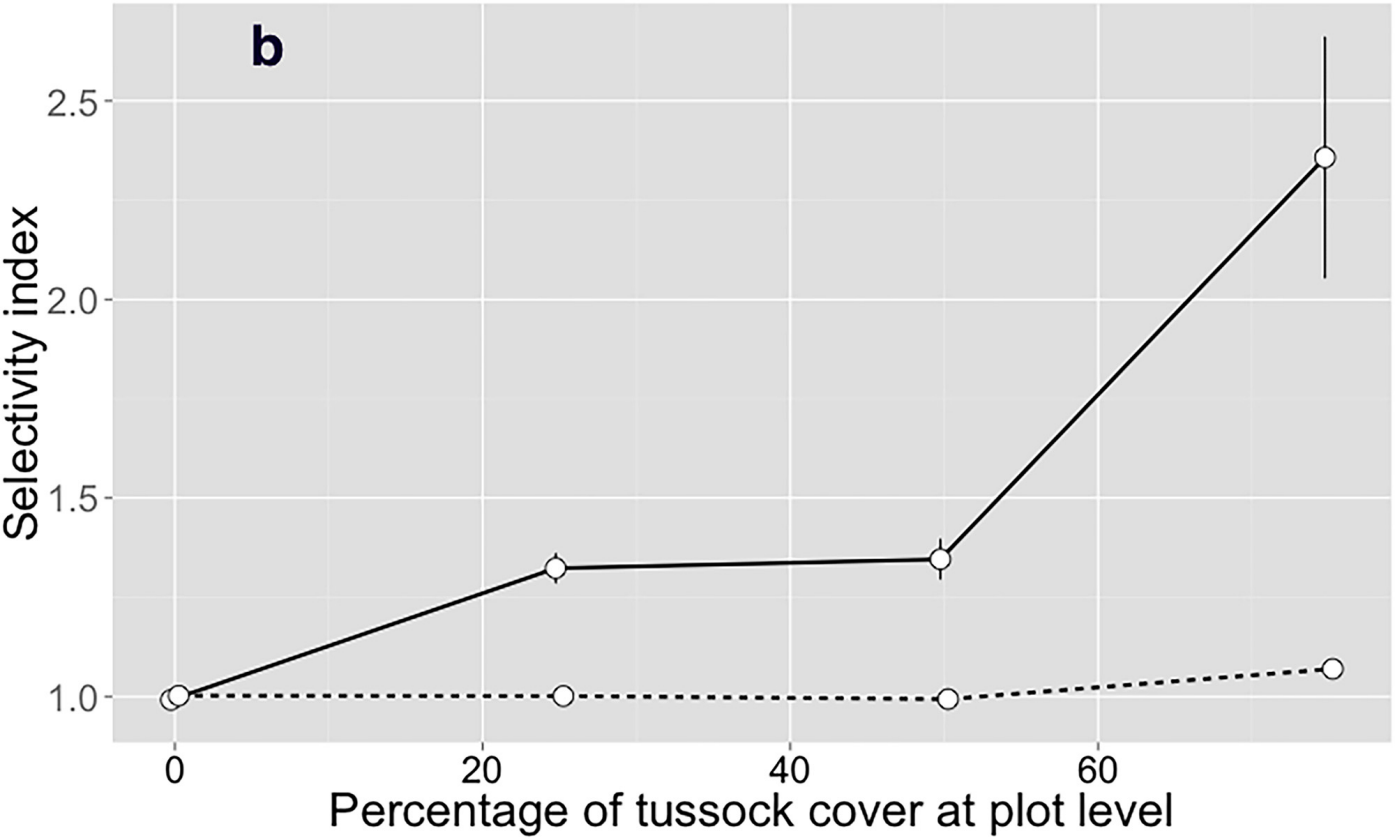
Partial selectivity levels (mean ± standard error) for inter-tussock stratum by ewes. The continuous line represents the feeding station to bite scale and the dashed line represents the search path or patch scale. Values >1.0 mean that the inter-tussock was selected more than expected by random encounter.

The interaction between selectivity scale and percentage of tussock at plot-scale was significant for selectivity of heifers (*P* = 0.0093; Table 6) and ewes (*P* < 0.001; Table 7). Neither heifers (Fig 5) nor ewes (Fig 6) exhibited any relevant selectivity for inter-tussock at the large selectivity scale (patch or plot level). The partial selectivity from feeding station to bite level by heifers and ewes increased nonlinearly with increasing percentage of tussock cover except for heifers between treatments with 25 and 50% tussock cover. Ewes were more selective than heifers, particularly at high proportions of tussock cover (Tables 6 and 7 and Figs 5 and 6).

**Table 6:** F tests for terms in the model of selectivity of heifers as a function of plot-level treatment and scale of selectivity.

Effect	Degrees of freedom	F	P(>F)
% Tussock at plot-scale (T)	3	1.428	0.2488
Scale (S)	1	43.051	0.0730
Interaction S x T	3	25.101	0.0093

**Table 7:** F tests for terms in the model of selectivity of ewes as a function of plot-level treatment and scale of selectivity.

Effect	Degrees of freedom	F	P(>F)
% Tussock at plot-scale (T)	3	5.229	0.0022
Scale (S)	1	43.051	< .0001
Interaction S x T	3	25.101	< .0001

## Discussion

Overall, results show that both sheep and cattle tend to avoid the tussocks of the invasive species, with sheep being more selective than cattle. At low cover of *E*. *plana* animals consume this invasive plant without strong avoidance, but at high levels of invasion both animal species strongly avoid the invasive grass. Animals exhibited a clear diet preference curve that is the opposite to a “prey switching” curve. Switching means that the relative attack on a prey increases faster than the prey's relative abundance [13]. In simple terms, switching should tend to stabilize prey abundance, but the inherent avoidance of *E*. *plana* by the main grazers in the ecosystem, particularly sheep, is expected not only not control the invasion, but likely to promote it.

### Diet switching by cattle and sheep

In spite of the fact that herbage mass per unit area in the preferred and higher quality inter-tussock was not limiting to either species, both species exhibited a nonlinear increase in tussock grazing as the proportion of area covered by tussocks increased. This response was observed at both the small (1 m^2^) and large (plot) scales, whereby the effect of increases in tussock cover in the small scale was stronger when large-scale tussock cover was greater (Fig 2), with the exception that responses were almost identical in the 50 and 75% cover treatments.

These results provide valuable insight into the role of horizontal heterogeneity on foraging strategies and on the dynamics of typical pastures and grasslands that exhibit a similar architecture of interspersed tall, low quality and short, high quality patches. Typical analyses of foraging strategies of ruminants have focused in the within-patch herbage density and quality [35, 36, 37, 38, 39]. Strategies used by mammalian herbivores to respond to changing horizontal proportions of different patch types received much less attention and are the focus of this discussion. Because of their lower absolute energy requirements, smaller ruminants (sheep) should outcompete larger ones (cattle) in short swards resembling the inter-tussock [23, 40]. [41] confirmed the importance of sward height or mass of the preferred sward component in determining the level of off take of non-preferred vegetation.

The analysis of both probability of grazing tussock and selectivity is necessary because these variables represent different aspects of the plant-animal interaction. In this paper, probability of grazing tussock is a proxy for the proportion of tussock in the diets of grazers. This proportion is relevant to derive potential consequences for the plant community and animals. Proportion of tussock in diets is one of the components that determine how much tussock mass is removed per unit time and areas. The other components are intake per animal and animal density. Proportion of tussock is also a component in the nutritional quality of diets and thus a determinant of animal growth and reproduction. However, proportion of a forage in the diet does not indicate if animals prefer or avoid the plant. Selectivity or electivity indices such as the one we used are used to determine if plants are avoided or preferred. In the experiments we found that although proportion of tussock in grazer diets increased with increasing proportion available, proportions in the diet were always lower than available and indicated a strong avoidance of tussocks at the feeding site to bite scale.

The present question is: what are the strategies for herbivores when abundance within tussock and inter-tussock are set and they face varying proportions of interspersed short and tall patches? This is a key question because most native grasslands and pastures exhibit such structure when grazed continuously at sustainable stocking rates. The “within-patch” theory, based on quantity and quality of available herbage, would not predict any changes in behavior as proportion of tussocks increases, for as long as within tussock and inter-tussock herbage remains the same. Yet we observed differential responses in both animal species. Increasing tussock abundance resulted in reduced intake rate [29]. Given that inter-tussock herbage was not limiting, why did animals change their behavior? And why were changes different between animal species? To answer these questions, we need consider the overall nutritional consequences of changing proportions of patch types.

Several hypotheses can explain the pattern of increased consumption of tussocks. We list them in order of increasing complexity. First, increased proportion of tussock means that tussock encounters will be more frequent unless there is an effort to avoid them by selection at the patch level. However, such selectivity were not observed (Figs 5 and 6). Thus, a constant probability of biting the herbage encountered will lead to linearly related increases in proportion of tussocks and tussock consumption. Although this mechanism cannot be ruled out, other mechanisms must have operated, because the change in probability of tussock grazing was not linearly related to proportion of tussocks (Figs 2 and 3). Second, increasing tussock proportion may have imposed a restriction to access to the inter-tussock with an associated reduction in bite mass, which motivated animals to increase bite rate [29] by incorporating more tussock in the diet through reduced selectivity for inter-tussock at the feeding station to bite level (Figs 5 and 6). This idea is supported by [42] and [43], who argued that the greatest potential for selection appeared to exist in the choice of feeding stations. Third, foraging theory suggests that animals would have evolved the ability to monitor the rate of energy or nutrient intake as a function of behavior, particularly selection among easily identifiable and effected choices, such as tussock and inter-tussock. Presumably, selectivity was set to obtain high intake rates of net nutrient and energy [44, 45, 46, 47, 48, 49, 50, 51].

The differential response of ewes and heifers is explained by the allometry of intake and nutrient requirements. Both cattle and sheep had a lower preference for tussock than inter-tussock vegetation, and cattle grazed tussocks more frequently than sheep (Figs 2 and 3), as previously reported [52, 41, 22, 53]. Ewes had the ability to selectively graze legumes from inside tussocks and exhibited greater probability of intra-tussock grazing as proportion of tussocks increased (Fig 4). Sheep have narrow jaws that allow them to target small plant parts with high nutritional value [53]. This greater ability to be selective allowed sheep to avoid the invasive grass more than cattle.

According to [20], the differences in scales of selectivity among different herbivores could be a mechanism driving niche separation, because patches that are not utilized by larger herbivores due to insufficient size or resource density (e.g. intra-tussock vegetation), may contain several smaller patches with sufficient resource density to be selected by smaller herbivores. Ewes were more selective than heifers at the smaller scale of perception evaluated (integrated from feeding station to bite level; Fig 6), when the short patches were limited (up 50% of tussocks). According to [54], when sheep encounter forage with marked heterogeneity in quality, selective grazing for high-quality components is their primary strategy and, thereby, they avoided the tussocks (Fig 3; [52, 55, 56, 57]).

We found that there are large differences in the way the two main grazers of the Campos Biome adjust their grazing behavior and diet selection to increasing abundance of an invasive alien grass. These differences may be crucial to design grazing methods that deter the invasion. Diet selection is one of the main mechanisms through which grazing animals influence plant communities [58]. Grazing impact on grassland plant species richness differs between sheep and cattle [59, 60, 17, 61, 9]. Sheep grazing may promote the invasion of *E*. *plana* in grasslands, since the preferred species—inter-tussock vegetation—must compete for growth resources against tall dominant species that are not incurring the same defoliation costs [62]. Moreover, sheep maintain high selectivity for inter-tussock herbage even when there is a large proportion of area covered by tussocks. The stabilizing effects of diet switching are not realized, and the invasive tussock species benefit. On the other hand, invasion by *E*. *plana* may be slower under cattle grazing. According to [61], both the type of grazer and grazing intensity have to be carefully adjusted to local conditions in order to achieve beneficial results of grazing for biodiversity. Our results indicate that neither sheep nor cattle would control the invasion because they would not significantly graze the tussocks of the invasive grass until the area is heavily infested. Therefore, traditional grazing methods with large pastures and continuous grazing will promote invasion by avoided grasses like *E*. *plana*, such as it has been seen in South American grasslands [15]. Mowing could be an alternative to grazing, but it would not create the additional structural heterogeneity promoted by selective grazing [10, 63] that is vital to promote floral and faunal diversity [58, 64, 65].

## Supporting Information

S1 Database Data base for heifers percent of bites and selectivity at smaller scale. (TXT)

S2 Database Data base for ewes percent of bites and selectivity at smaller scale. (TXT)

S3 Database Data base for heifers selectivity at path scale. (TXT)

S4 Database Data base for ewes selectivity at path scale. (TXT)

S5 Database Data base for sward characteristics for heifers. (TXT)

S6 Database Data base for sward characteristics for ewes. (TXT)

S1 Rcode R codes for heifers percent of bites and selectivity at smaller scale. (R)

S2 Rcode R codes for ewes percent of bites and selectivity at smaller scale. (R)

S3 Rcode R code for heifers selectivity at path scale. (R)

S4 Rcode R code for ewes selectivity at path scale. (R)

S5 Rcode R code for sward grazed by heifers. (R)

S6 Rcode R code for sward grazed by ewes. (R)
